# 

**Published:** 1903-12-12

**Authors:** 


### Publications.

Just Published. 8vo. with Photographic reproductions, 3/- net.

**THE PHYSIOGNOMY OF MENTAL DISEASES AND DEGENERACY.** By James Shaw M.D., Author of "Epitome of Mental Disease," etc., formerly Med. Supt. and Co-Licensee Haydock Lodge Asylum. Reprinted, with alterations and additions, from various articles in the "Medical Annual."

Just Published. Crown 8vo 1/6 net.

**A POCKET-BOOK OF CLINICAL METHODS.** By Chas. H. Melland, M.D.Lond., M.R.C.P., Physician to the Ancoats Hospital, Manchester.

THIRD EDITION. 12th Thousand. Small 8vo. Illustrated with 200 Original Drawings, 2/6. Lantern Slides of the Illustrations, 1/- and 1/6 each.

**"FIRST AID" TO THE INJURED AND SICK:** An Advanced Ambulance Handbook. By F. J. Warwick, B.A., M.B., and A. C. Tunstall, M.D., F.R.C.S.

THIRD EDITION. 2/- each, or 27/6 the Set of 16 Sheets, or mounted on linen, 45/- With nickel head.

**(Adopted by the War Office, Admiralty, and London School Board.)**

**LARGE SHEET "FIRST AID" DIAGRAMS:** Being Enlargements of the Illustrations in the above work on sheets 26 in. by 40 in., suitable for lectures and classes.

### MEDICAL ACCOUNTS.

Now is the time to arrange for this troublesome business, and

**WRIGHT'S THREE-BOOK SYSTEM,**
**WRIGHT'S SINGLE-BOOK SYSTEM,**
(10th Edition)
**WRIGHT'S VISITING LISTS, 1904,**
**WRIGHT'S CHARTS of all kinds,**
(Nearly 400,000 of which have been sold)
**WRIGHT'S CHART HOLDERS,**
(In use all over the world)
**WRIGHT'S CERTIFICATE BOOKS,**
**PROFESSIONAL ACCOUNT FORMS,**
**LETTER HEADINGS, and**
**DISPENSING LABELS.**

Are most of them better than the ordinary run of these things, and their use saves time and trouble.

PLEASE SEND FOR FREE SAMPLES.

Bristol: JOHN WRIGHT & CO. London: SIMPKIN & CO., Ltd.

JUST OUT. With 3 coloured Plates, and 54 other Illustrations. Price 6/- net.

### HANDBOOK OF DISEASES OF THE EAR

For Students and Practitioners.

By RICHARD LAKE, F.R.C.S. Eng., Surgeon to the Royal Ear Hospital.

London: BAILLIÈRE, TINDALL & COX, 8 Henrietta Street, Covent Garden.

### THE HANDBOOK OF THE OPEN-AIR TREATMENT

By Dr. Charles Reinhardt

(Visiting Physician to Hailey Sanatorium, Wallingford, and Stourfield Park Sanatorium, Bournemouth),

Contains Views, Descriptions, and Information respecting 30 Sanatoria in Great Britain.

Price: Cloth. 2/6; Paper, 1/- (postage 4d.)

The "HEALTH RESORT" Publishing Office, 379 Strand, London, W.C.

JUST ISSUED. EIGHTH EDITION. Crown 8vo. 10s. 6d.

### PHARMACY, MATERIA MEDICA AND THERAPEUTICS

is now rewritten and printed in large type, and is made a companion to the following volume. It contains an account of the action **of every drug in the B.P.**, as well as a section upon all the newest drugs, with an Introduction to the Art of Dispensing. With woodcuts, prescriptions, &c.

Also FOURTH EDITION. 1056 pages, 8vo. 16s.

### DICTIONARY OF TREATMENT.

Containing a Revised and up-to-date Article upon every Diseased Condition, whether Medical, Surgical, Gynæcological, or Ophthalmological, as well as Articles upon Poisoning, Drowning, and the various Surgical Emergencies and Accidents, arranged for Rapid Reference.

By W. WHITLA, M.A., M.D., Professor of Materia Medica, Queen's College, Belfast; Senior Physician to the Royal Victoria Hospital, &c.

The Publisher will supply both volumes, carriage paid, for 21/-

London: HENRY RENSHAW, 356 Strand.

FOURTH EDITION. Plates contain 93 Coloured and Stereoscopic Figures, 209 Illustrations in the Text. 17/6 net, including Stereoscope.

By P. WATSON WILLIAMS, M.D.(Lond.), Physician in Charge of Throat Department, Bristol Royal Infirmary, &c., &c.

### DISEASES OF THE NOSE, PHARYNX, AND LARYNX.

WITH A SECTION ON THE EXAMINATION OF

### THE EAR.

**Press Notices.**—"We know of no better practical guide"—*Practitioner.* "Certain to receive the recommendation of all teachers."—*Brit. Med. Journ.* "We can strongly recommend the book."—*Lancet.* "Excellent... ..The features of the work—completeness, clear description, and practical utility—are absolutely maintained in the new edition.—(Transn.)." *Internat. Centr. f. Laryngologie.*

Bristol: J. WRIGHT & CO. London: SIMPKIN & CO.

### WORKS BY Mr. McADAM ECCLES, M.S., F.R.C.S.,

Assistant Surgeon St. Bartholomew's Hospital, &c.

### HERNIA.

Second Edition, 1902. Price 7/6 net. Illustrated by Numerous Photographs.

### THE IMPERFECTLY DESCENDED TESTIS.

Just published. Profusely Illustrated. Price 7/6.

London: BAILLIÈRE, TINDALL & COX.

Crown 8vo. Illustrated. Price 2s. 6d. net.

### ANÆSTHETICS. By J. Blumfeld,

M.D.Cantab., Senior Anæsthetist to St. George's Hospital; Anæsthetist to the Grosvenor Hospital for Women and Children, London, and to the National Dental and Metropolitan Hospitals.

*Lancet.*—"....In writing this handbook the author has been quite successful." *Australasian Medical Gazette.*—"We can strongly commend this book." *Medical Chronicle.*—"The book is one which we can heartily recommend to senior students and practitioners."

London: Baillière, Tindall & Cox, 8 Henrietta St., Covent Garden.

### NOTICE TO CORRESPONDENTS.

All MSS., Letters, Books for Review, and other matters intended for the Editor should be addressed The Editor, "The Hospital," The Hospital Buildings, 28 & 29 Southampton Street, Strand, W.C.

The Editor cannot undertake to return rejected MS., even when accompanied by stamped directed envelope.

### Notice to Advertisers and Subscribers.

All Advertisements, Orders for Copies of The Hospital, and Business Communications should be addressed to **THE MANAGER (Not to the Editor),** The Hospital, 28 & 29 Southampton Street, Strand, London, W.C.

The Cost of Subscription, post free, is as follows:—Per week, 3½d.; per quarter, 3s. 9d.; per half-year, 7s. 6d.; per annum, 15s. Foreign and Colonial:—Per annum, 19s. 6d., payable, in all cases, in advance.

**NOTICE.—Vol. XXXIV. of "The Hospital" (April 1903 to September 1903), in handsome cloth binding, is now on sale at the office of this Journal, price 10s. 6d. Cases for binding the Numbers contained in this Volume 2s. Index to Vol. XXXIV., 2½d., post free.**

**The Monthly part for December is now ready. Price 1s., by post, 1s. 3d.**

Royal London Ophthalmic Hospital Guild.—The fourth annual meeting of the Royal London Ophthalmic Hospital Guild was held at 41 Bryanston Square, Mrs. William Lang, Chairman of Council of the Guild, presiding. The Guild, as stated in the report, undertakes to supply all clothing, bed-linen, and blankets required by the hospital, and it succeeded in raising £307 in 1902-3. Part of this sum was used for the endowment of an adult bed and a child's cot for one year, and it is sincerely hoped that enough will be subscribed in 1903-4 to enable the Guild to continue this branch of its work. The members of the Guild now number 170, with 98 associates, making a total of 268, of whom 56 joined during the past year.

### THE STUDENT OF MEDICINE.

### APPOINTMENTS.

**H. H. Ballachey**, L.R.C.P.Edin., M.R.C.S.Eng., Certifying Factory Surgeon for the Heckington District, Lincolnshire. **J. M. Bernstein**, M.B., M.R.C.S., Assistant Pathologist and Curator of the Museum to Westminster Hospital. **Joseph Buck**, L.R.C.P., L.R.C.S.Edin., L.F.P.S.Glasg., Medical Officer to the New Workhouse and Infirmary of the Hunslet Union, Rothwell Haigh, Leeds. **A. K. A. Caesar**, L.R.C.P.&S.Edin., L.F.P.S.Glasg., District Medical Officer of Health of the East Ashford Union. **H. Cardin**, M.R.C.S., L.R.C.P.Lond., District Medical Officer of the Chelmsford Union. **W. P. Cockle**, M.D., District Medical Officer of the Brentford Union. **W. Conway Gent**, L.R.C.P., L.R.C.S.Edin., Honorary Surgeon to the Fairford Cottage Hospital. **Clarence Haslewood**, M.B., C.M.Aberd., Medical Officer and Public Vaccinator for the Slaidburn District of the Clitheroe Union. **A. W. Jenkins**, M.B.Lond., M.R.C.S.Eng., Certifying Factory Surgeon for the Hinckley District, Leicestershire. **Eric C. Lindsay**, M.R.C.S., L.R.C.P.Lond., Assistant House Surgeon to the Scarborough Hospital and Dispensary. **Geoffrey Lucas**, B.A.Cantab., L.S.A.Lond., Assistant House Physician to the Westminster Hospital. **A. A. J. McNabb**, M.B., B.S.Durh., District Medical Officer of the Louth Union. **Charles Pye Oliver**, M.D.Lond., Physician to the West Kent General Hospital, Maidstone. **Charles Roberts**, M.B., B.S.Lond., F.R.C.S.Eng., Assistant Honorary Surgeon for Children to the Northern Hospital for Women and Children, Manchester. **T. Shanahan**, L.R.C.S.I., L.A.H.Dub., Certifying Factory Surgeon for the Kilmacthomas District, Waterford. **E. R. Thomas**, L.R.C.P.&S.Edin., L.F.P.S.Glasg., District Medical Officer of the Bangor and Beaumaris Union. **T. H. B. Yorath**, L.S.A., District Medical Officer of the Carmarthen Union

### "The Hospital" Institutional Library.

"Hospitals and Asylums of the World." 4 Vols., with a Portfolio of Plans, complete £12 12s.

"Burdett's Hospitals and Charities, 1903." 5s. net.

"Burdett's Official Nursing Directory, 1903." 3s. net.

"Cottage Hospitals, General, Fever, and Convalescent." 10s. 6d.

"Uniform System of Accounts for Hospitals and Public Institutions." New Edition. 4s. net.

"Hospital Expenditure: The Commissariat." 2s. 6d.

"A Legal Handbook for the Use of Hospital Authorities." 2s. 6d.

"The Cottage Hospital Case Book or Register of Patients." 10s. and 12s. 6d.

All these are published by the Scientific Press, Ltd., and may be obtained through any bookseller, or direct from the publishers, 28 and 29 Southampton Street, Strand, London, W.C.

### THE NURSING PROFESSION: How and Where to Train.

**By Sir HENRY BURDETT, K.C.B.**

**A reliable and up-to-date Guide to Training for the calling of a Nurse. In its pages would-be Nurses will find all the information they require.**

New Edition. Price **2/-**, post free **2/4.**

Of all Booksellers or of THE SCIENTIFIC PRESS, LTD., 28 & 29 Southampton Street, Strand, London, W.C.

**Hints on How to Start a District Nursing Association.**

BY A COUNTY SUPERINTENDENT.

Price 1/1 post free.

This work, which is the outcome of great personal experience, gives full particulars on how to start a District Nursing Association, and will be found of the greatest value to those who desire to establish such an institution in their own immediate neighbourhood.

The information it contains will also prove exceedingly useful to all in any way interested in District Nursing.

*Of all Booksellers, or of*

THE SCIENTIFIC PRESS, LTD., 28 & 29 Southampton Street, Strand, London, W.C.

### Two Useful Text-books.

**ELEMENTARY ANATOMY AND SURGERY FOR NURSES.**

By WILLIAM McADAM ECCLES, M.S. Lond., M.B., F.R.C.S., L.R.C.P.

Crown 8vo., profusely illustrated, cloth, **2/6** post free.

"The book will be valuable to nurses who are beginning the study of anatomy. The instruction is given very clearly and concisely, and the reader is able to glean much information in a very condensed form."—*Nursing Notes.*

**ELEMENTARY PHYSIOLOGY FOR NURSES.**

By C. F. MARSHALL, M.D., B.Sc., F.R.C.S.

Crown 8vo., illustrated, cloth, **2/-** post free.

"Nurses will find in it all that they require to know of the subjects treated."—*Daily Chronicle.*

*To be obtained of all Booksellers, or of*

THE SCIENTIFIC PRESS, LTD., 28 & 29 Southampton Street, Strand, London, W.C.

### THE EXAMINATION OF THE URINE.

By J. K. WATSON, M.D.Edin., M.B., C.M.,

Author of a "Handbook for Nurses." Bound in cloth boards, 1/- net; post free, 1/1.

### PRACTICAL GUIDE TO SURGICAL BANDAGING AND DRESSINGS.

By WM. JOHNSON SMITH, F.R.C.S.

Profusely illustrated, cloth, 2/- post free.

### NURSING NOTES ON MIDWIFERY AND GYNÆCOLOGY.

By SISTER ROSS, Rotunda Hospital, Dublin.

Cloth boards, 2/- net; 2/1 post free.

*Obtainable of all Booksellers.*

THE SCIENTIFIC PRESS, LTD., 28 & 29 Southampton Street, Strand, London, W.C.

### Useful Christmas Presents for Nurses.

### A HANDBOOK FOR NURSES.

By J. K. Watson, M.D., M.B., C.M.

Second Edition. Crown 8vo. over 400 pp, profusely illustrated, **5s.** net, **5s. 5d.** post free.

"A concise and comprehensive exposition of as much medical knowledge as a nurse needs. The work cannot but prove useful to those for whom it has been designed."
*Scotsman.*

### ELEMENTARY ANATOMY AND SURGERY FOR NURSES.

By William McAdam Eccles, M.S.Lond., M.B., &c.

Crown 8vo. Illustrated, cloth, **2s. 6d.** post free.

"Valuable to nurses who are beginning the study of anatomy. The instruction is given very clearly and concisely, and the reader is able to glean much information in a very condensed form."—*Nursing Notes.*

### NURSING: ITS THEORY AND PRACTICE.

By Percy G. Lewis, M.D., M.R.C.S., L.S.A.

New and Enlarged Edition.

Crown 8vo. over 400 pp. profusely illustrated, **3s. 6d.** post free.

"Nurses will find the book full of interest and instruction, which cannot fail to assist them in their work."
*British Medical Journal.*

### ELEMENTARY PHYSIOLOGY FOR NURSES.

By C. F. Marshall, M.D., B.Sc., F.R.C.S.

Crown 8vo. Illustrated, cloth, **2s.** post free.

"Nurses will find in it all that they require to know of the subjects treated."—*Daily Chronicle.*

### A PRACTICAL HANDBOOK OF MIDWIFERY.

By Francis W. Haultain, M.D.

New and Revised Edition. Illustrated. Handsomely bound in leather, **6s.** net, **6s. 3d.** post free.

"One of the best of its kind and well fitted to perform the functions its author claims for it."

*Edinburgh Medical Journal.*

### MIDWIVES' POCKET BOOK.

(With Chapter on the Midwives Bill.)

By Honnor Morten.

Small crown 8vo cloth, **1s.** post free.

An exceedingly useful handbook for those entering for the L.O.S. certificate as well as for qualified Midwives.

### SURGICAL WARD-WORK AND NURSING.

By Alexander Miles, M.D.Edin., C.M., F.R.C.S.E.

Revised Edition. Demy 8vo. Illustrated, cloth gilt, **3s. 6d.** net, **3s. 10d.** post free.

"An illustrated manual which will furnish much needed guidance to the student and the nurse in the early stages of their apprenticeship."—*The Times.*

### ART OF MASSAGE.

By A. Creighton Hale.

Second Edition. Demy 8vo. Profusely illustrated, cloth gilt, **6s.** post free.

"Mrs. Hale seems to have had considerable experience, not only as a practitioner, but as a teacher of the art, and explains in a series of clearly illustrated articles the many complaints for which massage has been found beneficial, and the special manipulations required in each case."
*Morning Post.*

### OPHTHALMIC NURSING.

By Sydney Stephenson, M.B., F.R.C.S.E.

Second Edition. Crown 8vo. illustrated, with glossary of terms, cloth, **3s. 6d.** net, **3s. 10d.** post free.

"May very advantageously be studied by nurses who are about to enter the Ophthalmic service of a hospital."
*Lancet.*

### ART OF FEEDING THE INVALID.

By a Medical Practitioner and a Lady Professor of Cookery.

New and Popular Edition. Demy 8vo. cloth, **1s. 6d.** post free.

"A new edition of this valuable work is very welcome, and we heartily commend it to all who desire reliable and practical instruction in sick cookery."—*Nursing Notes.*

### NURSING IN DISEASES OF THE THROAT, NOSE AND EAR.

By P. Macleod Yearsley, F.R.C.S.Eng., M.R.C.S., L.R.C.P.Lond.

Demy 8vo. Illustrated, cloth, **2s. 6d.** post free.

"Of practical service to every nurse who wishes to be thoroughly trained."
*Scottish Medical and Surgical Journal.*

### PRACTICAL GUIDE TO SURGICAL BANDAGING AND DRESSINGS.

By Wm. Johnson Smith, F.R.C.S.

Demy 16mo., profusely Illustrated (suitable for apron pocket), cloth, **2s.** post free.

*COMPLETE CATALOGUE POST FREE ON APPLICATION.*

The SCIENTIFIC PRESS, LTD., 28 & 29 Southampton Street, Strand, London, W.C.

### WORKS FOR NURSES.

**LESSONS ON MASSAGE.** By Mrs. MARGARET D. PALMER, Manager of the Massage Department and Instructor of Massage to the Nursing Staff of the London Hospital. Second Edition enlarged and revised; pp. xvi. + 262 with 118 Illustrations, plain and coloured. *Just Published.* Price 7/6 net.

**A MANUAL FOR STUDENTS OF MASSAGE.** By Miss M. A. ELLISON, L.O.S. With 50 original Illustrations. Price 3/6 net.

**A MANUAL FOR HOME NURSING.** By BERNARD MYERS, M.D., M.R.C.S., Surgeon and Lecturer to St. John's Ambulance Association. *Just Published.* Price 2/6 net.

**NOTES ON HOME NURSING.** By Miss D. M. GOLDIE, Lecturer on Nursing, Hygiene, &c., to the County Councils of London, Surrey and Middlesex. *Just Published.* Price 1/6 net.

**THE FEEDING AND HYGIENE OF INFANTS AND CHILDREN.** By JOHN McCAW, M.D., M.R.C.P., Physician to the Belfast Hospital for Children. *Just Published.* Price 2/6.

**DISEASES OF THE HAIR, INCLUDING GREYNESS, BALDNESS, &c., AND THEIR TREATMENT.** By DAVID WALSH, M.D., Western Hospital for Diseases of the Skin. Price 2/6 net.

**MENSTRUATION AND ITS DISORDERS.** By ARTHUR E. GILES, M.D., B.Sc., F.R.C.S., Surgeon Chelsea Hospital for Women. Price 2/6 net.

**MOVABLE ATLAS OF THE ANATOMY AND PHYSIOLOGY OF THE FEMALE GENERATIVE ORGANS AND OF PREGNANCY.** Second Edition. With Text. By ARTHUR E. GILES, M.D., F.R.C.S. Price 3/- net.

**MOVABLE ATLAS OF THE ANATOMY AND PHYSIOLOGY OF THE CHILD.** With Explanatory Text. By D'ARCY POWER, M.A.Oxon, M.B., F.R.C.S. Price 3/- net.

**BAILLIÈRE'S POPULAR MOVABLE ATLAS OF THE ANATOMY AND PHYSIOLOGY OF MAN.** Size 16 by $7\frac{1}{2}$ inches. With Explanatory Text. By W. S. FURNEAUX, Special Science Teacher London School Board. Price 3/6 net.

London: BAILLIÈRE, TINDALL & COX, 8 Henrietta Street, Covent Garden.

### To Nurses.

### A NEW CASE BOOK FOR PRIVATE NURSING.

**Arranged for Day and Night Nurse's Report, with rulings in accordance with what has been found in practice to be the most suitable and approved System for Pay Hospitals, Nursing Homes, Private Nurses, &c.**

DAY NURSE'S REPORT.

*Date*............

| Time | Nourishment | Quantity | Urine | Stimulants, Medicines, and Remarks |
|---|---|---|---|---|
| | | | | |

| Total Nourishment | Bowels | Summary |
|---|---|---|
| | | |

(Facsimile of ruling.)

**Price 3d. net.** Post free 4½d.

**The Size of an Exercise Book (7½ in. by 9½ in.).**

*Obtainable of all Booksellers.*

**Price 3d. net.** Post free 4½d.

*Special Quotations for a quantity can be procured from the Publishers.*

THE SCIENTIFIC PRESS, Ltd., 28 & 29 Southampton Street, Strand, London, W.C.

